# Prevalence of malaria, prevention measures, and main clinical features in febrile children admitted to the Franceville Regional Hospital, Gabon

**DOI:** 10.1051/parasite/2016032

**Published:** 2016-08-05

**Authors:** Sydney Maghendji-Nzondo, Hermann Nzoughe, Guy Joseph Lemamy, Lady Charlene Kouna, Irene Pegha-Moukandja, Faustin Lekoulou, Bertrand Mbatchi, Fousseyni Toure-Ndouo, Jean Bernard Lekana-Douki

**Affiliations:** 1 Unité de Parasitologie Médicale (UPARAM), Centre International de Recherches Médicales de Franceville (CIRMF) B.P. 769 Franceville Gabon; 2 Département de Parasitologie-Mycologie Médecine Tropicale, Faculté de Médecine, Université des Sciences de la Santé B.P. 4009 Libreville Gabon; 3 Département de Biologie Cellulaire et Génétique, Université des Science de la Santé B.P. 4009 Libreville Gabon; 4 Département de Biologie, Université des Sciences et Techniques de Masuku B.P. 901 Franceville Gabon

**Keywords:** Febrile syndrome, Preventive measures, *Plasmodium*, Species, Children, Gabon

## Abstract

Recently, major progress has been made in controlling malaria in Africa. However, in Gabon, little information is available on the role of malaria in childhood febrile syndromes, the use and efficacy of preventive measures, and *Plasmodium* species distribution. Here, we characterized malaria in febrile children in Franceville, Gabon through a cross-sectional study at the pediatric unit of the Franceville Regional Hospital. We registered 940 febrile children. Their general condition was markedly altered in 11.7% of cases (*n* = 89/760); among them 19 (21.4%) had a severely altered condition. Malaria was the second most frequent etiology (22.0%; *n* = 162/738), after respiratory tract infections (37.3%; *n* = 275/738). Children with malaria (63 ± 39 months) were older than children without malaria (40 ± 37 months) (*p* = 0.0013). Hemoglobin, red blood cell, white blood cell, and platelet values were lower in children with malaria than in those without malaria (*p* < 0.0001). Anemia was the most common feature of severe malaria (70.6%; *n* = 12/17), followed by neurological involvement (23.5%; *n* = 4/17). The prevalence of malaria was significantly higher in children older than 60 months than in younger children (40% vs. 15.5%; *p* < 0.0001). *Plasmodium falciparum* accounted for 97.5% of cases (158/162), followed by *Plasmodium malariae* (2.5%; *n* = 4/162). Bed net use was high (74.4%; *n* = 697/936) and contributed to malaria prevention (*p* = 0.001). Good basic knowledge of malaria also had a preventive effect (*p* < 0.0001). The prevalence of malaria in children in Franceville did not decrease significantly from 2009 to 2012, remaining at about 20%, highlighting that preventive measures should be reinforced.

## Abstract (fr)

Récemment, des progrès importants ont été accomplis dans la lutte contre le paludisme en Afrique. Cependant, au Gabon, peu d’informations sont disponibles sur le rôle du paludisme dans les syndromes fébriles des enfants, l’utilisation et l’efficacité de mesures de prévention, et la répartition des espèces de *Plasmodium*. Dans ce travail, nous avons caractérisé le paludisme chez les enfants fébriles à Franceville, au Gabon par une étude transversale à l’unité pédiatrique de l’Hôpital régional de Franceville. Nous avons enregistré 940 enfants fébriles. Leur état général était nettement altéré dans 11.7 % des cas (*n* = 89/760), et sévèrement altéré chez 19 d’entre eux (21.4 %). Le paludisme était la deuxième étiologie la plus fréquente (22.0 % ; *n* = 162/738), après les infections des voies respiratoires (37.3 % ; *n* = 275/738). Les enfants atteints de paludisme étaient plus âgés (63 ± 39 mois) que les enfants sans paludisme (40 ± 37 mois) (*p* < 0.005). L’hémoglobine, les globules rouges, les globules blancs et les valeurs plaquettaires étaient plus faibles chez les enfants atteints de paludisme que chez ceux sans paludisme (*p* < 0.0001). L’anémie est la caractéristique la plus commune du paludisme grave (70.6 % ; *n* = 12/17), suivie des atteintes neurologiques (23.5 % ; *n* = 4/17). La prévalence du paludisme était significativement plus élevée chez les enfants âgés de plus de 60 mois que chez les enfants plus jeunes (40 % contre 15 % ; *p* < 0.0001). *Plasmodium falciparum* représentait 97.5 % des cas (158/162), suivie par *Plasmodium malariae* (2.5% ; *n* = 4/162). L’utilisation des moustiquaires était répandue (74 % ; *n* = 697/936) et a contribué à la prévention du paludisme (*p* = 0.001). Une bonne connaissance de base du paludisme a également un effet préventif (*p* < 0.0001). La prévalence du paludisme chez les enfants de Franceville n’a pas diminué de façon significative de 2009 à 2012, demeurant à environ 20 %, ce qui souligne que les mesures préventives doivent être renforcées.

## Introduction

Febrile syndromes are one of the main reasons for pediatric consultations in developing countries and may be due to various viral, bacterial, and parasitic infections. Malaria is the most widespread febrile disease, present in 99 countries and territories. One-third of the world population is at risk, and malaria causes about one-fifth of all childhood deaths worldwide [13]. In 2013, there were an estimated 198 million cases of malaria and 584,000 deaths [42].

Five *Plasmodium* species infect humans, namely *falciparum*, *vivax*, *malariae*, *ovale* [33], and *knowlesi*, an emerging species [36]. *Plasmodium falciparum* is the most widespread and most lethal species in Africa, causing 95% of symptomatic cases [33].

New antimalarial policies implemented in recent years have mainly concerned *P. falciparum*, although *P. vivax* and *P. knowlesi* can also cause life-threatening malaria [14, 19, 34]. *Plasmodium knowlesi* can cause cerebral hemorrhage, lung heaviness, and cardiac hemorrhage [6, 7]. Premature reticulocyte death due to *P. vivax* infection can cause severe anemia over a period of several months by blocking the formation of mature red blood cells [1].

Gabon is hyperendemic for malaria. Transmission is perennial because the equatorial climate favors mosquito proliferation and larval development. Changes in the national antimalarial policy, based on artemisinin combination therapy (ACT), distribution of impregnated bed nets, and intermittent preventive treatment during pregnancy, have led to a decline in the malaria burden in urban areas. Between 2005 and 2008, malaria prevalence dropped significantly from 31.2% to 18.3%, followed by a recrudescence in 2011 in Libreville (24.1%). In Franceville, malaria prevalence dropped from 69% (2004) to 19.5% (2009). Between 2010 (17.9%) and 2012 (21.45%), no significant change was observed [2, 17, 20]. Artemether-lumefantrine (AL) and artesunate-amodiaquine (ASAQ) are both drugs in the co-first lines of treatment. Only three human species (*P. falciparum*, *P. malariae*, and *Plasmodium ovale*) have been reported in Gabon [21, 31]. The most prevalent is *P. falciparum* (94–99%), followed by *P. malariae* (0.5–5%) and *P. ovale* (0.5–2.4%). The near absence of human *Plasmodium vivax* infection in Central and Western Africa has been attributed to a low prevalence of the Duffy receptor in these populations [24]. However, cases of *P. vivax* infection have been described in Duffy (-) subjects in Uganda and Central Africa [8, 23, 29]. Surveillance of all malaria species will be necessary to eradicate the disease.

The aim of this study was to determine the place of malaria in febrile children consulting at the pediatric unit of the Franceville Regional Hospital, Gabon, as well as its characteristics and the efficacy of preventive measures.

## Materials and methods

### Ethics statement

The study was approved by the Gabonese National Ethics Committee and the Ministry of Health (N° 00370/MSP/CABMD). Blood samples were collected with the parents’ or guardians’ informed consent.

### Study location and population

We conducted a cross-sectional study between April 2011 and May 2012 in the pediatric unit of the Amissa Bongo Regional Hospital in Franceville, the capital of the Haut-Ogooué Province, Gabon. Children between 6 and 168 months of age presenting an axillary temperature >37.5 °C or a >24-hour history of fever were recruited.

Global health alteration was classified as severe in the presence of anorexia, asthenia, and weight loss, and moderate in the presence of two of these symptoms. The alteration was defined as uncomplicated in the presence of only one of these three symptoms. Weight loss in the child was estimated by using the last weight recorded in the health book.

### Recording of preventive measures

Data on the use of bed nets and insecticides, and some information on education and knowledge of malaria were collected with a questionnaire (Supplementary Material). The parents/guardians were asked whether their children slept under bed nets and whether they sprayed insecticide in their houses. To determine their level of education on malaria, we asked the following questions: “Do you know malaria? What is malaria? Do you know the modes of transmission of malaria? How can you avoid malaria?” Correct responses to these questions were considered a basic knowledge of malaria.

### Diagnosis

The OptiMAL-IT^®^ rapid diagnostic test (RDT) was used [27]. Parasite load was determined on blood smears using the Lambaréné method [30]. This method of counting slides is a variant of a method for counting thick films. Ten microliters of blood is evenly distributed on a 10- by 18-mm area of a microscope slide. Each high-power field (HPF) on this thick smear is 1/500th of a microliter (on a standard microscope at 1,000 magnification), and a count is made per 10 HPFs. The parasitemia per microliter is calculated by a precalibrated appropriate multiplication factor (500). Children with RDT or positive blood smears were considered to have malaria. Respiratory tract infections corresponding to colds and coughs, gastroenteritis corresponding to diarrhea and/or vomiting, dermatosis, ear, nose, and throat (ENT) infections, acute algetic syndromes, as well as clinical symptoms, such as pale conjunctivae and global health state alteration (anorexia, asthenia, and weight loss), were diagnosed by a physician according to clinical classification.

### Hematological analyses

Routine hematological assays were performed with an automated blood cell counter (STKS^®^, Coulter Corporation, USA). Blood (5 mL) was collected in EDTA tubes. Plasma was stored at −20 °C and blood pellets were used for DNA extraction. Moderate anemia was defined by a hemoglobin level between 5 and 10 g/dL, and severe anemia by a hemoglobin level ≤5 g/dL.

### DNA extraction

DNA from all children was extracted with the Omega Bio-Tek E.Z.N.A.1 method (Omega Bio-Tek, USA) according to the manufacturer’s protocol [16]. Briefly, 250 μL of blood pellets, 25 μL of OB protease (20 mg/mL), and 250 μL of lysis buffer were mixed and heated to 65 °C for 30 min before adding 260 μL of isopropanol. The mixture was transferred to a column and centrifuged at 10,000 rpm for 1 min. The column was washed twice at 13,000 rpm for 2 min, and DNA was eluted with 90 μL of sterile water preheated to 65 °C. DNA samples were kept at −20 °C until use.

### Identification of *Plasmodium* species by RFLP-PCR

*Plasmodium* speciation was based on nested PCR amplification of the *cytochrome b* gene, followed by enzymatic restriction, as previously described [39]. Five microliters of DNA was amplified with 1X Taq polymerase buffer (Invitrogen), 0.8 μM of each primer (Plas 1 and 2a for primary PCR and Plas 3 and 4 for nested PCR), 0.2 mM dNTP (Invitrogen), 2 mM MgCl_2_, and 0.024 U of Taq DNA polymerase (Invitrogen), using the following cycling program: 5 min at 94 °C, followed by 35 cycles of 30 s at 94 °C, 45 s at 58 °C, 45 s at 72 °C, and a final extension step for 7 min at 72 °C. PCR products were detected by 2% agarose gel electrophoresis. In order to distinguish the four species of *Plasmodium* (*falciparum*, *malariae*, *ovale*, and *vivax*), restriction fragment length polymorphism (RFLP) analysis was performed using the restriction enzyme *AluI* (New England Biolabs, UK). PCR primers and *Plasmodium* species profiles after *AluI* enzyme digestion are shown in Table 1. DNA products were detected by electrophoresis on 2% agarose gel.

**Table 1.** Sequences of primer sets and restriction fragments for each species.

Gene		Primers	*T* (°C)	Sizes of PCR products after *Alu I* restriction (bp)			
				*P. falciparum*	*P. malariae*	*P. ovale*	*P. vivax*
*Cytochrome b*	PCR 1	5′-GAGAATTATGGAGTGGATGGTG-3′	58	640		584	
		5′-GTGGTAATTGACATCCWATCC-3′			381		
	PCR 2	5′-GGTGTTTYAGATAYATGCAYGC-3′			249	224	249/270
		5′-CATCCWATCCATARTAWAGCATAG-3′		159	187		187/169/111

### Statistical analysis

Statistical analyses were carried out with Epi-info version 3.3.2 (2005, CDC, Atlanta, USA) and STATA version 10 (Stata Corp, College Station, USA). Age was expressed as the median and interquartile range (IQR), and parasite density as the geometric mean (GMPD). The Chi-square test was used to compare categorical variables, and the non-parametric Mann-Whitney *U* test, Pearson’s test, and Fisher’s exact test were used for group comparisons, as appropriate. *p* values <0.05 were considered to indicate statistical significance.

## Results

### Study population

A total of 940 children were included: 556 in 2011 and 384 in 2012. The sex ratio was 1.1 (489M/451F). Ages were recorded for only 931 children. Children aged between 6 and 60 months represented 72.6% (676/931) of the patients, and children over 60 months 27.4% (255/931). The general characteristics of the children are summarized in Table 2. Mean body temperature was 38.3 ± 1.1 °C. Hematological values were normal.

**Table 2.** General characteristics of the included patients.

	Mean ± standard deviation
Parameters	
Age (months)	45.2 ± 38.8
Temperature (°C)	38.3 ± 1.1
Hemoglobin (g/dL)	10.7 ± 2.1
White blood cells (/μL)	10,197 ± 6,337.4
Red blood cells (/μL)	3,852,112.7 ± 806,732.2
Platelets (/μL)	286,054.8 ± 157,151.7

Clinical examination showed that the global health state was altered in 11.7% (89/760) of children, among these 78.6% (70/89) were moderate and 21.4% (19/89) severe. Conjunctival examination in 728 children showed a normal aspect in 235 cases (32.3%), a pale conjunctival aspect in 451 cases (61.9%), and a reddish aspect in 42 cases (5.8%).

**Figure 1.**
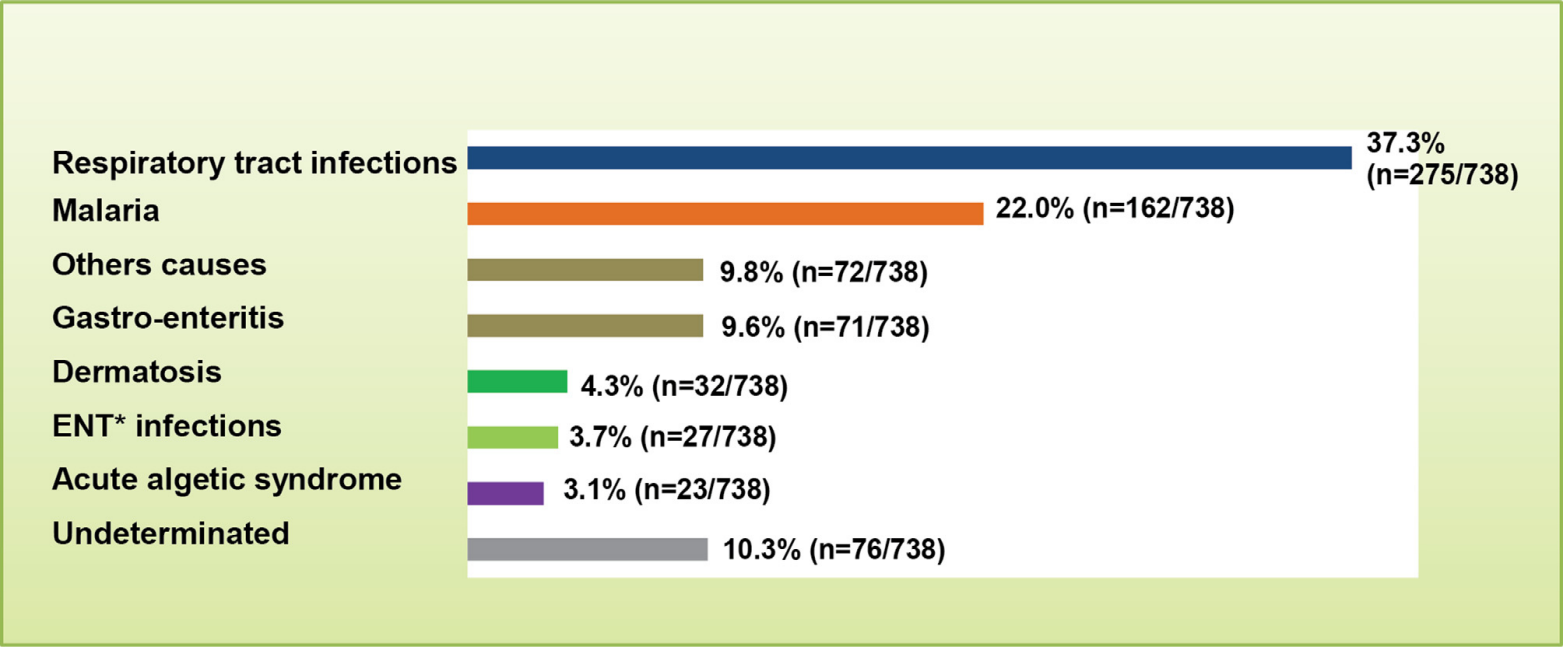
Distribution of certain fever diseases diagnosed in the children included in the study. *Ear, nose, and throat infections.

The causes (etiology) of fever were investigated in 738 cases (Fig. 1). The most prevalent cause was a respiratory tract infection (37.3%; *n* = 275), followed by malaria (22.0%; *n* = 162). Gastroenteritis and other causes were found in 9.6% (*n* = 71) and 9.8% (*n* = 72) of cases, respectively. Dermatoses, ENT infections, and an acute algetic syndrome were responsible for fever in 4.3% (*n* = 32), 3.7% (*n* = 27), and 3.1% (*n* = 23) of cases, respectively. The cause of fever was undetermined in 10.3% of cases (*n* = 76).

### Effect of malaria on hematological parameters and age distribution

The mean age of the *Plasmodium*-infected patients (63.4 ± 39.4 months) was significantly higher than that of the other children (40.3 ± 37.1 months) (*p* = 0.0013), (Table 3). The body temperature of the *Plasmodium*-infected children (38.5 ± 1.1 °C) was higher than that of the other children (38.2 ± 0.9 °C) (*p* = 0.001). Hemoglobin, red blood cell, white blood cell, and platelet values were lower in *Plasmodium*-infected children than in uninfected children (*p* < 0.0001). The mean interval between symptom onset and consultation was 3 days in both *Plasmodium*-infected and uninfected children.

**Table 3.** Comparison of characteristics between *Plasmodium* uninfected versus infected children (diagnosed by RDT and blood smear).

	Mean ± standard deviation		*p*
	Uninfected (*N* = 731)	Infected (*N* = 209)	
Age (months)	40.3 ± 37.1	63.4 ± 39.4	0.001
Temperature (°C)	38.2 ± 0.9	38.5 ± 1.1	0.001
Hemoglobin (g/dL)	11.0 ± 1.8	9.6 ± 2.6	4.95 × 10^−18^
White blood cells (×10^3^/μL)	10.8 ± 6.3	8.6 ± 6.4	7.93 × 10^−6^
Red blood cells (×10^6^/μL)	3.9 ± 0.7	3.5 ± 0.9	2.27 × 10^−18^
Platelets (×10^3^/μL)	321.6 ± 144.6	188.6 ± 147.4	1.47 × 10^−29^
Parasitemia (geometric mean and quartile)	9,581 (50–600,000)		

### Characteristics of plasmodial infection

**Figure 2.**
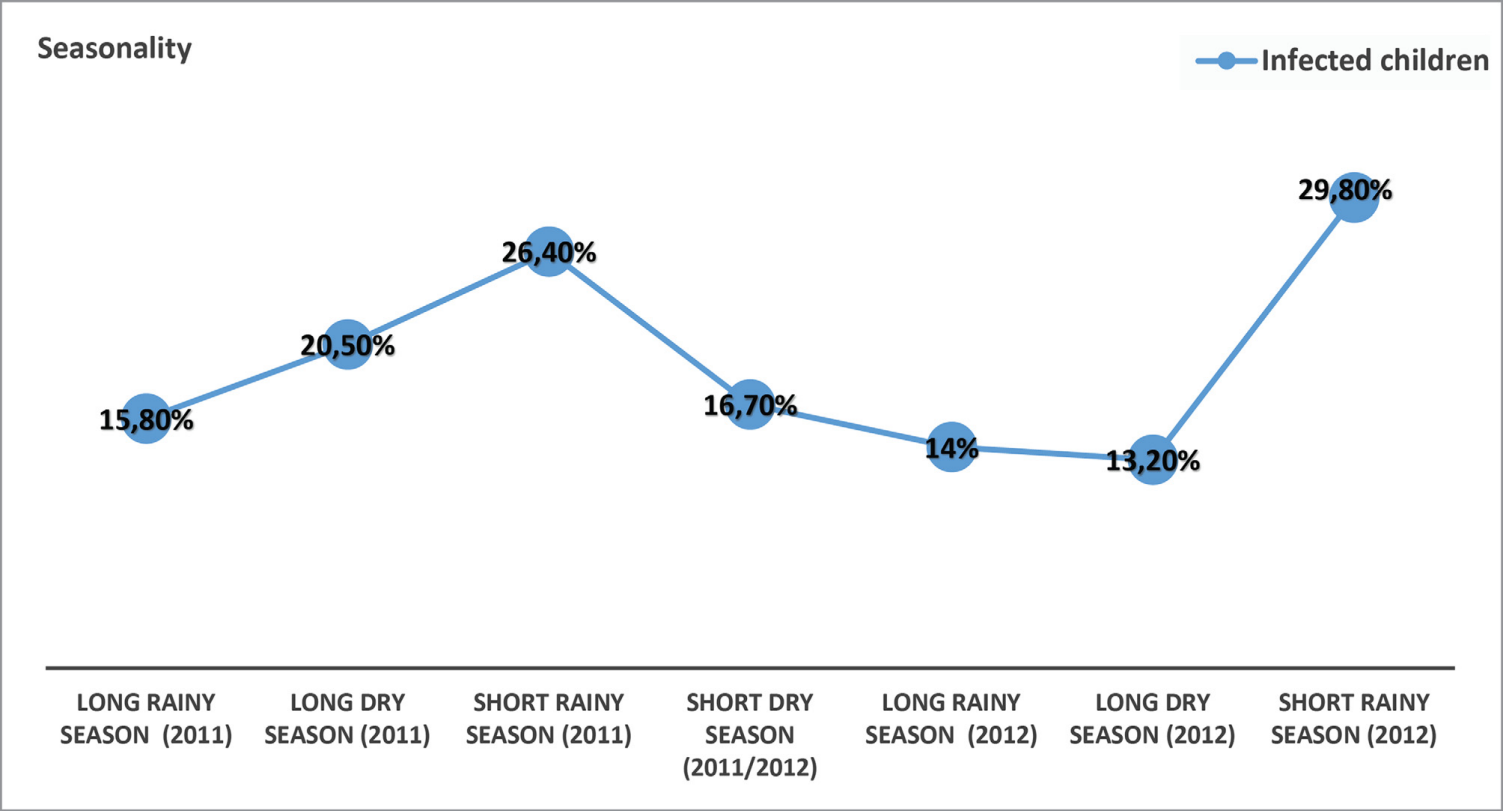
Temporal distribution of plasmodial infection.

The overall prevalence of *Plasmodium* infection was 22.2% (*n* = 209/940) by RDT and 18.8% on blood smear examination (*n* = 166/881) (*p* = 0.08). The sex ratio was 1.2 (115M/94F) in the *Plasmodium-*infected group and 1.1 (374M/355F) in the uninfected group (*p* = 0.4). The mean values of parasitemia were calculated based on the 166 samples that tested positive by thick film only. Mean *Plasmodium* parasitemia was 9581 (50–600,000) p/μL of blood. The prevalence of malaria was similar in 2011 [23% (*n* = 128/556)] and 2012 [21.1% (*n* = 81/384)] (*p* = 0.5). The temporal distribution of the parasite infection with the seasons shows that overall prevalence of *Plasmodium* infection was not the same depending on the season in 2011–2012 (*p* = 0.02) (Fig. 2). The prevalence of *Plasmodium* infection was significantly lower during the long rainy season in 2011 (15.8%), and during the long rainy season (14%) and long dry season (13.2%) in 2012. It was highest during the short rainy seasons (29.8% and 26.4%) in 2012 and 2011 (*p* < 0.04).

Among the *Plasmodium*-infected children, 8.1% (17/209) had one sign of severe malaria. Severe anemia was the most prevalent sign [70.6% (12/17)], followed by neurological involvement [23.5% (4/17)]. There was one case of prostration (5.9%).

The maximum age among the *Plasmodium*-infected children was 172 months. The prevalence was significantly lower in children under 60 months of age [15.5% (105/676)] than in children over 60 months [40% (102/255)] (*p* = 2.4 × 10^−15^) (Table 4). The prevalence was lower in children under 12 months of age [2.5% (5/198)] than in the other age groups (*p* < 0.00001). The prevalence was similar in children over 108 months of age (36.5%) and in those between 37 and 108 months [(40.4%; *n* = 147/364 (*p* = 0.7)]. The prevalence was significantly lower in the 13–36 months age group [17.5% (55/315)] than in older age groups (*p* < 0.05), and significantly lower from 37 to 60 months [27.6% (45/163)] than from 61 to 84 months [40.8% (42/103)] and from 85 to 108 months [43.3% (29/67)] (*p* = 0.02). Mean parasitemia was significantly lower in children aged 37–60 months [7,421 (200–160,000) p/μL] than in those aged 61–84 months [16,832 (200–600,000) p/μL], (*p* = 0.04). Parasitemia was also significantly higher in children aged from 61 to 84 months, and lowest in children over 108 months [5,804 (1,000–600,000) p/μL], (*p* = 0.01). Finally, we observed a significant difference in mean parasitemia between children aged 85 and 108 months [22,971 (1,000–340,000) p/μL] and those over 108 months [7,139 (1,000–125,000) p/μL], (*p* = 0.02).

**Table 4.** Prevalence of malaria infection according to age group in children (*n* = 931) (children diagnosed by RDT and blood smear).

	Age group (months)					
	6–12	13–36	37–60	61–84	85–108	>108
Prevalence %, (*n*/*N*)	2.5, (5/198)	17.5, (55/315)	27.6, (45/163)	40.8, (42/103)	43.3, (29/67)	36.5, (31/85)
Parasitemia (p/μL)	1,854 (50–143,000)	18,624 (1000–446,333)	12,132 (300–160,000)	24,065 (200–600,000)	27,091 (1000–340,000)	7,139 (1000–125,000)

%: percentage; *n*: number of infected children; *N*: number of recruited children.

### Distribution of *Plasmodium* species

Molecular diagnosis was based on amplification of an 816 bp fragment of the *Cytb* gene. Speciation was based on enzymatic digestion (RFLP) of PCR products. *Plasmodium falciparum* was characterized by two DNA fragments (640 and 159 bp), and *P. malariae* by three fragments (187, 249, and 381 bp). Only 162 PCR products could be digested, because of insufficient yield and the low sensitivity of the primer set used. We found that 158 (97.5%) infections were due to *P. falciparum,* and 4 (2.5%) to *P. malariae*. No mixed infections and no *P. ovale*, or *P. vivax* infections were found.

### Preventive measures

Finally, we analyzed the patients’ sociodemographic characteristics and use of preventive measures (Table 5). We found that bed nets were used by 74.5% of patients (697/936). The frequency of bed net use was significantly higher among children without malaria [76.9%, *n* = 561/729] than among children with malaria [65.7%, *n* = 136/207], (*p* = 0.001). The use of window nets [14.7%, *n* = 137/935] had no preventive effect (*p* = 0.4), and neither did the use of insecticide house spraying [respectively, 29.6% (216/729) and 22.7% (47/207)] in children without and with malaria (*p* = 0.06). Insecticide sprays were used in 28.1% of homes (*n* = 263/936). The parents/guardians of children with and without malaria were aware of essential information on malaria in, respectively, 51% (*n* = 105/207) and 71.3% (*n* = 519/728) of cases (*p* = 4.8 × 10^−8^).

**Table 5.** Preventive measures between malaria infected and uninfected patients.

	Number of children			
Prevention measures	Uninfected	Infected	*p*	Odds ratio CI 95%
Bed net	561 (76.9%)	136 (65.7%)	0.001	1.74 [1.2–2.4]
Insecticides	216 (29.6%)	47 (22.7%)	0.06	1.4 [1–2.05]
Window net	111 (15.3%)	26 (12.6%)	0.4	1.3 [0.8–1.9]
Receiving Information, Education and Knowledge (IEK) on malaria	519 (71.3%)	105 (51%)	4.8 × 10^−8^	2.4 [1.7–3.3]

## Discussion

Elimination or eradication of malaria is a new major challenge in some endemic areas. Herein, we assessed the epidemiology of malaria in febrile children in South-East Gabon (Franceville). More than three-fourth of febrile children reporting to the Franceville Regional Hospital during the study period had an altered global health state, probably linked to a high prevalence of anemia, as indicated by the large proportion of children with conjunctival pallor. Similar results have been obtained in Tanzania [25]. We confirm that children aged less than 5 years are the most likely to present fever [37], and that infectious diseases account for fever in 80% of these children. Low hemoglobin levels are due to parasite-induced hemolysis [3]. Indeed, *P. falciparum* antigens, such as RSP2/RAP2, could be transferred to the surface of uninfected and infected red blood cells, reducing their deformability, inducing their sequestration by the spleen, accelerating both complement-mediated lysis as well as macrophage uptake, leading to anemia [11]. *Plasmodium falciparum* infection also modulates the cytokine balance, leading to erythrocyte clearance [26]. However, malnutrition and digestive parasitic infections are also causes of anemia [15, 44].

In addition, we confirm that malaria is the second leading cause of fever among children in Franceville, after respiratory tract infections [4, 17]. Gastroenteritis was the third most common cause of fever, as also reported in Tanzania, where malaria was the second leading cause of fever [9]. Although we did not explore the pathogens responsible for respiratory tract infections, a recent report from Gabon (including Franceville) showed that the most prevalent are influenza-like viruses (adenoviruses, parainfluenza viruses, enteroviruses, respiratory syncytial viruses, and influenza viruses) [18]. The cause of fever remained undetermined in 10% of children, underlining the shortcomings of the Gabonese healthcare facilities.

Our findings confirm that malaria mainly affects older febrile children in Gabon [20]. However, our findings are based only on children in consultation at our study sites; we do not have information on malaria distribution in the overall population. Children under 5 years are more often protected by bed nets and other preventive measures than older children whose prevention measures are often unavailable. We found an overall high rate of bed net use. Insecticide-treated bed nets have been shown to significantly reduce the incidence, severe forms, and lethality of malaria, especially among children under five years old. As in several other African countries, the use of bed nets has contributed to the decline in the malaria burden in Gabon [40]. It should be noted that information on the use of bed nets in this study was based on the verbal statements of parents/guardians, and that bed net condition (presence of holes, insecticide impregnation) was not verified.

We found that children under seven years of age tended to have lower parasite loads. The same result has been reported after the increased coverage of insecticide-treated bed nets and the decrease of malaria transmission in some countries [38, 43]. However, parasitemia tended to be lower after 108 months of age, a finding consistent with acquisition of semi-immunity.

The low frequency of malaria in children less than 12 months is consistent with maternal immune protection through breast-feeding. The higher prevalence of malaria among febrile children over 5 years is consistent with reports from Rwanda [32], where the risk was highest in the 5- to 15-year-old age group. A similar age distribution has been reported in asymptomatic Nigerian children [28]. This could suggest a delay in immunity acquisition, because of the use of preventive measures that limit the contact between children and the parasite. Children aged 5 to 15 years could thus represent a reservoir for *Plasmodium*. Surprisingly, the data indicate that malaria prevalence was highest in the short rainy seasons. Indeed, breeding sites in this season are maintained while they are cleaned in long rainy seasons.

We confirm the decrease in the malaria burden in Franceville in recent years, despite its stability since 2009 [17]. In contrast, however, a recrudescence has been reported in febrile children up to 24.1% in Libreville, capital of Gabon, after the decline observed between 2000 and 2009 [5, 20]. This could be explained by different socioeconomic contexts and/or plasmodial transmission rates, and by a decrease in free distribution of impregnated bed nets and awareness campaigns on malaria. Since the year 2008, Gabon has lost a global grant.

Hematological values were lower in *Plasmodium*-infected children than in other febrile children. This is consistent with red blood cell lysis and platelet sequestration induced by *P. falciparum*, as recently reported in India [35]. We confirm that malaria is also associated with a loss of white blood cells [22]. Severe malaria anemia was found in 70.6% of our patients with severe malaria, consistent with data from Libreville, Gabon [12].

*Plasmodium falciparum* was responsible for the majority of cases of malaria in this study, as reported elsewhere in Gabon [21, 31]. This suggests that, despite the decrease in the malaria burden in Gabon, the species distribution in febrile children has not changed. In contrast, among asymptomatic individuals living in rural areas of Gabon, it was recently reported that *P. malariae* and *P. ovale* accounted for, respectively, 47.6% and 9.9% of cases [10]. This could suggest selection of *P. falciparum* to the detriment of other species during acute malaria. Another possible explanation is the use of different diagnostic methods. Delicat-Loembet et al used 454 sequencing to identify *Plasmodium* species, while we and other authors used less sensitive methods (PCR-RFLP, and blood smear with RDT, respectively). Despite the non-detection of *P. ovale* in our study, this species is occasionally found in Franceville, and we have diagnosed one case of *P. ovale* infection since the end of this study. The low sensitivity of the PCR can be explained by insufficient yield and the low sensitivity of the primer set used. We confirm that *P. vivax* does not circulate in humans in Franceville, and neither does *P. knowlesi*, a primate species that causes some episodes of severe malaria in humans [41].

We found that 66.7% of parents/guardians had a good knowledge of malaria, and confirm that this knowledge is associated with a lower risk of childhood malaria. Surprisingly, the use of window nets and insecticide sprays was not associated with a lower risk of malaria, possibly because few parents/guardians used these two measures.

We confirm the stability of the malaria burden in Franceville, Gabon, implying that stronger preventive measures are needed to reduce it further. Studies are also needed to evaluate the malaria burden throughout Gabon.

## Conflict of interests

The authors declare no conflict of interest in relation with this paper.
